# Letter to the Editor: New Values for Silicon Reference Materials, Certified for Isotope Abundance Ratios

**DOI:** 10.6028/jres.099.016

**Published:** 1994

**Authors:** P. De Bièvre, S. Valkiers, H. S. Peiser

**Affiliations:** Institute for Reference Materials and Measurements^1^, Commission of the European Communities, Joint Research Centre, B-2440, Geel, Belgium; National Institute of Standards and Technology, Gaithersburg, MD 20899-0001

**Keywords:** absolute abundances, atomic weights, cyclotron frequencies, isotope abundances, isotope ratio mass spectrometry, isotope reference materials, Penning trap, relative atomic masses, reference material, silicon, silicon tetrafluoride

## Abstract

New isotope abundance and relative atomic mass (atomic weight) values — with low, hitherto unattained uncertainty — are reported for two previously described silicon reference materials using a well-known method with an improved isotope-ratio mass spectrometer. These new values are directly traceable to the SI, more specifically to the unit for amount of substance, the mole, and independent of the SI unit of mass and of the Avogadro constant. Besides the residual mass-spectrometric uncertainties, these new values depend in effect only on a recently published direct comparison of the cyclotron frequency in a Penning trap of ^28^Si^+^ with that of ^12^C^+^.

Two new silicon isotope reference materials (RMs) have previously been announced [1]: IRM-017^2^ in chips of a silicon crystal (distributed in samples of about 2 mmol) and IRM-018^2^ in the chemical form of SiO_2_ (distributed in samples of about 0.1 mol) [2]. An independent set of absolute measurements with an improved mass spectrometer [3] is now reported. Some further refinement of the gas mass-spectrometric measurement has been achieved. In that method SiF_3_^+^ ions generated from a synthetic mixture of highly enriched specimens of Si isotopes are compared with these ions from the RMs of proven internal homogeneity [4]. Improved data analysis has in the meantime also been developed and adopted for all "standard atomic weight" evaluations by the Commission on Atomic Weights and Isotopic Abundances of the International Union of Pure and Applied Chemistry [5].

The uncertainties of the measurements here described still depend mostly on the mass-spectrometrically derived isotope abundance ratios. However, these uncertainties now approach those of the best values of the relative atomic masses of the silicon isotopes. For these masses we have based our calculations on the recent direct comparison in a Penning trap of the cyclotron frequency of ^28^Si^+^ with that of ^12^C^+^ [6]. The derived ^28^Si mass is uncertain by only 1 × 10^−8^ and only 3 × 10^−9^ higher than the value recently published by Audi and Wapstra [7] with a marginally lower estimated uncertainty by taking other measurements into consideration. Uncertainties in recognized values for the atomic masses of ^29^Si and ^30^Si are negligible because of their low abundances in terrestrial silicons.

Table 1 summarizes the new results. Their significance rests on:

1. the reduction of the uncertainties by one order of magnitude;
2. the direct traceability of these values to the mole, the SI unit for amount of substance, involving only relative mass measurements of the enriched samples, without any absolute mass measurement based on the kilogram;
3. the ability with these RMs to compare reliably silicon specimens at the 10^−7^ level of relative atomic mass (atomic weight) *A*_r_(Si), a level at which many geological sources and processed materials can be differentiated;
4. the reliable intercomparisons of these isotopic RMs with Si SRM 990 of NIST [8] enable the reduced uncertainty to be transferred to the latter without much loss. A value of *A*_r_(Si) = 28.085 538 ±0.000 018 might be indicated compared with the NIST certified value from 1975 measurements of *A*_r_(Si) = 28.085 526 ±0.000 056.

## Figures and Tables

**Table 1:** Isotopic composition and *A*_r_(Si) for both RMs. Expanded uncertainties, *U*, are indicated under the digits to which they relate and are computed on a two standard deviation basis.

Abundance ratio	IRMM-017		IRMM-018	
	New values	Previous values [1]	New values	Previous values [1]
*n*(^29^Si)/*n*(^28^Si)	0.050 771 5 6 6	0.050 69 12	0.050 844 2 4 8	0.050 83 12
*n*(^30^Si)/*n*(^28^Si)	0.033 488 9 7 8	0.033 52 10	0.033 585 1 6 6	0.033 60 10

Amount (of substance) Fraction				
^28^Si	0.922 287 7 8 6	0.922 33 14	0.922 144 0 7 0	0.922 14 14
^29^Si	0.046 825 9 5 8	0.046 75 11	0.046 885 7 4 2	0.046 88 11
^30^Si	0.030 886 4 7 0	0.030 92 8	0.030 970 3 5 8	0.030 98 8

Relative mean atomic mass (atomic weight) *A*_r_(Si)	28.085 408 15	28.085 40 19	28.085 635 12	28.085 65 19

## Footnotes

2 After the renaming of the Institute, these are now labelled IRMM-017 and IRMM-018.
